# Effect of Hyperglycemia on COVID-19 Outcomes: Vaccination Efficacy, Disease Severity, and Molecular Mechanisms

**DOI:** 10.3390/jcm11061564

**Published:** 2022-03-12

**Authors:** Celestino Sardu, Raffaele Marfella, Francesco Prattichizzo, Rosalba La Grotta, Giuseppe Paolisso, Antonio Ceriello

**Affiliations:** 1 Department of Advanced Medical and Surgical Sciences, University of Campania Luigi Vanvitelli, Piazza Luigi Miraglia 2, 80138 Naples, Italy; raffaele.marfella@unicampania.it (R.M.); giuseppe.paolisso@unicampania.it (G.P.); 2 Mediterranea Cardiocentro, 80122 Naples, Italy; 3 IRCCS MultiMedica, Via Fantoli 16/15, 20138 Milan, Italy; rosalba.lagrotta@multimedica.it (R.L.G.); antonio.ceriello@hotmail.it (A.C.)

**Keywords:** SARS-CoV-2, diabetes, glycemic control, COVID-19, vaccination, inflammation, cardiovascular complications, myocarditis, COVID-19 therapy, COVID-19 prognosis

## Abstract

Background/Aims: The severe acute respiratory syndrome coronavirus 2 (SARS-CoV-2) is a positive-stranded single-stranded RNA virus, a member of the subgenus *Sarbecovirus* (beta-CoV lineage B) and responsible for the coronavirus disease 2019 (COVID-19). COVID-19 encompasses a large range of disease severity, from mild symptoms to severe forms with Intensive Care Unit admission and eventually death. The severe forms of COVID-19 are usually observed in high-risk patients, such as those with type two diabetes mellitus. Here, we review the available evidence linking acute and chronic hyperglycemia to COVID-19 outcomes, describing also the putative mediators of such interactions. Findings/Conclusions: Acute hyperglycemia at hospital admission represents a risk factor for poor COVID-19 prognosis in patients with and without diabetes. Acute and chronic glycemic control are both emerging as major determinants of vaccination efficacy, disease severity and mortality rate in COVID-19 patients. Mechanistically, it has been proposed that hyperglycemia might be a disease-modifier for COVID-19 through multiple mechanisms: (a) induction of glycation and oligomerization of ACE2, the main receptor of SARS-CoV-2; (b) increased expression of the serine protease TMPRSS2, responsible for S protein priming; (c) impairment of the function of innate and adaptive immunity despite the induction of higher pro-inflammatory responses, both local and systemic. Consistently, managing acute hyperglycemia through insulin infusion has been suggested to improve clinical outcomes, while implementing chronic glycemic control positively affects immune response following vaccination. Although more research is warranted to better disentangle the relationship between hyperglycemia and COVID-19, it might be worth considering glycemic control as a potential route to optimize disease prevention and management.

## 1. Introduction

Severe Acute Respiratory Syndrome Corona Virus 2 (SARS-CoV-2) has been spreading across the world since December 2019. Corona Virus Disease 2019 (COVID-19), the disorder caused by SARS-CoV-2, can present as a highly heterogenous acute condition, ranging from patients with mild respiratory symptoms to severe or critical pneumonia [1,2]. In critical cases, a significant number of subjects experience organ dysfunction (acute cardiac and kidney injury), acute respiratory distress syndrome, and eventually death. Various possible factors, e.g., age and different comorbidities, have been suggested as major determinants of different disease severity, albeit the exact molecular mechanisms leading to divergent outcomes are still a matter of investigation [3,4]. In other studies, type 2 diabetes mellitus (T2DM) has been suggested as a risk factor for poor COVID-19 prognosis [5,6,7,8]. Patients with T2DM often present a plethora of comorbidities, e.g., cardiovascular diseases, obesity, and metabolic syndrome, which it has been suggested may mediate the observed increased risk for complicated COVID-19 [5,6,7,8,9]. However, hyperglycemia *per se* has been suggested not only to be a potential mediator of increased disease severity but also of blunted immune response to COVID-19 vaccination.

## 2. Aims of the Review

The aim of this review is to synthesize: (a) the literature showing that acute and chronic hyperglycemia are both linked to a poor prognosis in COVID-19; (b) the putative molecular mechanisms linking hyperglycemia and insulin resistance to an increased SARS-CoV-2 pathogenicity; (c) the possible modifying effect of hyperglycemia on COVID-19 therapies; and (d) the effect of glycemic control on the efficacy of vaccination against SARS-CoV-2. Then, we finally propose glycemic control, both acute and long-term, as a potential means to minimizing the deleterious consequences of SARS-CoV-2 infection and to maximize the effect of COVID-19 vaccine.

## 3. Literature Selection

We search PubMed using the following combinations of keywords: “COVID-19” OR “SARS-CoV-2” AND “hyperglycemia”, “COVID-19” OR “SARS-CoV-2” AND “diabetes”, “COVID vaccine” OR “SARS-CoV-2 vaccination” AND “glycemic control”, “COVID therapy” AND “hyperglycemia”. Collected manuscripts were non-systematically scrutinized for their content and were included if relevant for the scope of the narrative review.

## 4. Hyperglycemia Worsens the Prognosis of Patients with COVID-19

Hyperglycemia per se has been suggested as a risk factor that worsens the prognosis in COVID-19 patients with as well as without T2DM [10]. In a clinical setting, recent studies reported that T2DM diagnosis [11,12,13] and acute hyperglycemia at hospital admission [10,14] could negatively affect the clinical outcomes of patients with COVID-19. Indeed, hyperglycemia in COVID-19 is a strong predictor of worsening prognosis and mortality [14]. Therefore, early glycemic control has been suggested as a suitable therapeutic option to reduce the poor outcomes in hospitalized hyperglycemic COVID-19 patients with or without a previous diabetes diagnosis [15]. The increase of glycemia is accompanied by an over-production of inflammatory molecules and cellular mediators implied in pro-thrombotic processes, promoting the development of acute cardiovascular complications [14,15]. Thus, early glycemic control during the first phases of COVID-19 might improve clinical outcomes [10,14,15,16]. Notably, hyperglycemia in people with diabetes at the time of hospital admission is a more relevant risk factor than long-term glycemic control evaluated by glycated hemoglobin (HbA1c) values [10,14,15,16,17]. Similarly, an increased gap between admission glucose and HbA1 values is a relevant variable predicting mortality in critically ill patients with diabetes in an intensive care unit (ICU) [10,14,15,16,17]. Broadly, long-term glycemic control, measured as HbA1c, is associated with higher COVID-19 mortality [18]. In addition, a drop in glucose levels between admission and after 24 h from hospitalization is associated with a decreased incidence of progression to severe disease and death at 20 days, as observed in hyperglycemic patients with or without diabetes [15]. Intriguingly, hyperglycemia at hospital admission seems to be more relevant for COVID-19 prognosis in people without diabetes compared with patients with this condition [10,14,15,16,17]. Epidemiological data suggest that acute hyperglycemia occurs in about 50% of patients hospitalized for COVID-19, while the prevalence of diabetes in the same population is about 7% [17].

The origin of hyperglycemia in patients with COVID-19 is being investigated and is likely the result of multiple components. Indeed, SARS-CoV-2 might induce β-cell dysfunction, either directly by promoting a direct insult to this cell type or by inducing an increase of circulating pro-inflammatory cytokines which promote cell death. In addition, the induced inflammatory response might foster or aggravate insulin resistance, considering that soluble cytokines are known to interfere with the insulin pathway [14,19,20]. In this respect, the pathways and imbalances instigated by SARS-CoV-2 largely overlap with those already altered by obesity, diabetes, and metabolic syndrome [14,19,20]. Indeed, BMI is associated with a peculiar immune signature that foresees severe COVID-19 [21] and it has been suggested as a major driver of disease severity especially in younger patients [22]. Of note, obesity has been suggested as an “accelerator” of the aging of the immune system [23], a key mechanism determining COVID-19 outcomes [24]. Similarly, insulin resistance might synergize with the stress response induced by SARS-CoV-2 to affect cumulatively the course of the disease [25]. However, given the common clustering of multiple risk factors in patients with diabetes, it is difficult to establish if one component among obesity, insulin resistance, and hyperglycemia, is a more relevant driver of COVID-19 severity compared with the others.

Whether hyperglycemia in severe COVID-19 patients without diabetes is the result of an acute, critical inflammatory condition or whether it stems from the effect of SARS-CoV-2 infection in multiple, diabetes-relevant tissues is matter of investigation [19,20]. One interesting hypothesis suggests that hyperglycemia is the result of the damage caused by the virus to the mitochondrial machinery in multiple tissues [26]. Of note, the resulting Warburg effect has been proposed to promote viral replication [27]. Whatever the case, available data suggest that hyperglycemia often accompanies patients with severe COVID-19, independently of previous diabetes diagnosis, and that it represents a relevant prognostic factor for the relative clinical outcomes [17].

## 5. Hyperglycemia and COVID-19 Therapy

Clinical management of COVID-19 in patients with poor glycemic control is currently a work in progress [28]. The challenge is to provide for these patients the best anti-viral treatment, the right anti-diabetic medications, and optimized infusion therapies to have a proper control of in-hospital acute hyperglycemia [11,28]. Indeed, the goal is to find the best drug therapy for patients with T2DM to avoid hyperglycemia and its complications [10,11,12,13,14,15,16,17,28,29,30,31,32,33,34,35,36,37,38].

SARS-CoV-2 has been shown to alter the expression of glucose transporters (GLUT), thus deregulating cellular metabolism [39,40]. Indeed, immune phenotyping of patients with severe COVID-19 revealed an altered expression of GLUT1 in CD8+ T cells, as well as in intermediate and nonclassical monocytes [39]. In addition, it has been proposed that a decreased GLUT1/NHE1 RNA expression ratio in whole blood might predict disease severity in patients with COVID-19 [40].

The choice of anti-diabetic drugs might also influence the course of COVID-19, albeit no data are available at present to suggest a preferential use of one particular class [19,41,42]. Selected hypoglycemic agents, such as metformin, could play a protective role against COVID-19 via significant reduction of the inflammatory burden, though this effect might derive from a confounding-by-indication bias [42,43,44,45]. Similarly, there are no conclusive data regarding the effects exerted by other oral hypoglycemic drugs, such as the sodium-glucose transporter 2 inhibitors (SGLT2-I), the glucose-like peptide-1 (GLP-1) receptor agonists, and pioglitazone in humans with COVID-19 [41,45,46].

Independently of ongoing therapy, emerging and old data suggest that it is of utmost importance to closely monitor glycemia in cases of ICU admission, ensuring stable metabolic compensation and avoiding the associated over-inflammation [10,28,38,41]. In this context, it has been suggested that glycemic control in hospitalized patients and in those admitted to ICU should be handled by intravenous insulin, possibly using exact dosing with a perfusion device [10,28,38,41]. While insulin infusion has been shown to reduce mortality in COVID-19 patients [10], the use of continuous glucose monitoring (CGM) should also improve glucose variability, a phenomenon linked to an increased risk of death in ICU settings, also independently of COVID-19 and diabetes [8,47]. At least three studies have substantiated the usefulness of CGM in terms of feasibility, accuracy, meaningful reduction in the frequency of glucose testing, and possibly also in terms of increased time-in-range in COVID-19 patients in the ICU [48,49,50]. However, whether CGM improves hard outcomes in COVID-19 patients is unknown. 

Glycemic control can also influence the effect of other COVID-19 therapies. Indeed, hyperglycemia denied the beneficial effect of tocilizumab, a monoclonal antibody blocking the effect of IL-6, in terms of mortality reduction in COVID-19 patients. [28]. Of note, patients presenting with hyperglycemia were those with the highest levels of IL-6 [28], reinforcing the notion that hyperglycemia and inflammation are closely intertwined. To this respect, diabetes itself is a well-characterized source of inflammatory cytokines [51,52,53], while also acute hyperglycemia per se is sufficient to induce cytokine elevation in plasma [54]. Thus, it is reasonable to hypothesize that, albeit high IL-6 and glycemia levels might both simply sense an increased severity of COVID-19, the pro-inflammatory effect of glycemia could eventually hamper the effect of anti-inflammatory biologicals. While preliminary data have been reported for tocilizumab, no data relevant to this aspect are available for antibodies targeting other cytokines, e.g., the anti-IL-1 anakinra [42].

Overall, these data suggest the acute control of glycemia in patients with severe COVID-19 might help to improve hard outcomes, possibly through the modulation of viral spreading, the improvement of metabolic compensation, and a reduced inflammatory status, which could in turn also eventually positively interact with the effect of anti-inflammatory drugs [55,56].

## 6. Hyperglycemia Promotes SARS-CoV-2 Ligand to Human Cells, Its Intracellular Entrance, and Replication

SARS-CoV-2 infection also targets the endothelium [2] and, in case of severe infection, causes multi-organ failure, resulting in a hyper-inflammatory response and eventually acute thrombosis [11]. These mechanisms are initiated by the binding of SARS-CoV-2 to ACE2 receptors [13], followed by the entrance of SARS-COV-2 into host cells by cleavage of the S protein operated by the serine protease TMPRSS2, which generate the S1 and S2 subunits [13]. The relevance of ACE2 as the main binding protein for SARS-CoV-2 was demonstrated by in vitro experiments showing the binding of a synthetic receptor binding domain (RBD) to ACE2, an effect observed in absence of other putative targets such as dipeptidyl peptidase 4 (DPP4) [29]. Similarly, TMPRSS2 has variable expression and regulation in different human endothelial cell types and has been identified as a valid therapeutic target by specific non-coding-RNA approaches [12]. The S protein is cleaved into two subunits (S1 and S2) and the S1 subunit is further split into SA and SB domains, with the SB domain being the one held to bind human ACE2 [31]. The S2 subunit mediates the fusion of the virus-ACE2 complex with the cell membrane. Of note, such subunit has been suggested to be highly glycosylated at evolutionarily conserved sites [31,32].

Experimental evidence derived from cryo-electron microscopy suggests that the viral spike protein is a trimer and that one of the trimer RBD sites is exposed to bind ACE2 [30]. Additional structure–function studies also indicate that the viral spike S protein of SARS-CoV-2 is highly glycosylated [31]. Thus, it might be hypothesized that alterations in the glycosylation levels of both the spike protein and ACE2 can modulate viral binding and that such modifications could eventually modulate S protein–ACE2 complex fusion with the cell membrane. In addition, ACE2 expression could show an under- or an over-expression in patients with different clinical characteristics, particularly those with T2DM and hyperglycemia [13]. Indeed, hyperglycemia could promote SARS-CoV-2 pathogenicity via direct effects on the expression and activation of the ACE2 receptor [13]. In addition, hyperglycemia could lead to an altered expression of the different molecular pathways implied in the intra-cellular pathogenesis of SARS-CoV-2 in humans [13,14,15]. In this context, a recent work studied the effects of hyperglycemia in ex-vivo cardiomyocytes from explanted hearts of COVID-19 patients, showing that hyperglycemia induced a direct modification of the ACE2 oligomerization state, likely due to a glucose-induced mild glycation [13]. In addition, the same work showed an increased expression of glycated ACE2 in heart samples derived from T2DM compared with non-T2DM patients [13]. Of note, this process also affected the binding properties of the ACE2 monomer to the spike protein, supporting the occurrence of changes in the oligomerization process towards the dimer formation [13]. These processes induced by hyperglycemia might promote a higher susceptibility to SARS-CoV-2 infection since the oligomerization augments the binding affinity to spike [13,33,34]. In addition, hyperglycemia could enhance the SARS-CoV-2 transition from the pre-fusion state to the post-fusion in COVID-19 patients [13,33,34,35]. Finally, COVID-19 patients with hyperglycemia also showed an increased expression of TMPRSS2 [13]. The recruitment of TMPRSS by SARS-CoV-2 in human cardiomyocytes in hyperglycemic conditions has also been suggested as a potential cause for the observed high rate of myocardial damage in COVID-19 patients [13,33,34,35].

Overall, such evidence could suggest that hyperglycemia might promote SARS-CoV-2 replication and spreading through multiple mechanisms involving ACE2 and TMPRSS.

## 7. Hyperglycemia Promotes Inflammatory Endothelial Dysfunction and Myocarditis in COVID-19 Patients

Multiple mechanisms might explain the association between hyperglycemia and worst outcomes, including cardiac damage, in patients with COVID-19 [36]. SARS-CoV-2 shows a higher tendency to colonize the epithelium of the upper airways in asymptomatic patients and patients with mild clinical manifestations [2,37]. However, ACE2 is a receptor ubiquitously expressed in humans, particularly in the endothelium and other target organs [37]. In addition, SARS-COV-2 can infect liver tissue, brain tissue, and also cardiomyocytes [13]. Notably, cardiac injury is present in 24.4% of hospitalized COVID-19 patients, and about half of hospitalized COVID-19 patients with T2DM developed some degree of myocardial damage [13]. In this context, beyond its effect on viral spread, it has been suggested that hyperglycemia could impair innate and adaptive immunity, also fueling the exaggerated inflammatory response called “cytokine storm” [13,14,42]. Indeed, hyperglycemia has been suggested to increase the expression of cytokines, including IL-6 and tumor necrosis alpha (TNFα), in patients with severe COVID-19 [14,15,16]. A possible intermediate mechanism between hyperglycemia and the cytokine storm is oxidative stress. Indeed, it is recognized that hyperglycemia induces a plethora of alterations in the redox homeostasis and that many of those imbalances foster the activation of pro-inflammatory pathways [57,58]. Given the known effect of pro-inflammatory cytokines on the cardiac structure, such effect might increase SARS-CoV-2 pathogenicity and promote cardiac damage in patients with COVID-19, possibly contributing to the high rate of pericarditis, arrythmias, and myocarditis observed in COVID-19 patients [13].

SARS-CoV-2 can also exert direct pathogenic effects on the coronary endothelium [13,38]. Indeed, SARS-CoV-2 infection could cause dysfunction of the coronary endothelium with the over-activation of inflammatory and thrombotic processes [38]. These events could lead to an acute coronary syndrome, by the acute thrombotic occlusion of coronary vessels with consequent myocardial infarction [38]. Notably, SARS-CoV-2 positive patients evidenced higher thrombus burden and more severe alterations of coronary flux than did their negative counterparts [38]. In addition, SARS-CoV-2 particles have been be isolated from the coronary endothelium and the surface of coronary thrombi [38]. However, the isolation of SARS-CoV-2 from the thrombi of the coronary bed might stem from intra-coronary or systemic over-inflammation and thrombosis caused by the virus [38]. Remarkably, such observations have also been obtained in SARS-CoV-2 positive, asymptomatic patients [38].

Overall, these data suggest that COVID-19 is often accompanied by cardiac damage and that this effect might be further enhanced by metabolic perturbation at the cellular level [26] and by ambient hyperglycemia, either through its ability to promote SARS-CoV-2 spreading or by feeding the cytokine storm, a phenomenon suggested to mediate a large effect of the disease and the development of several complications.

## 8. Vaccination against SARS-CoV-2: Effects in Patients with Diabetes and Hyperglycemia

An almost worldwide and intense vaccination campaign has partly limited the spread of SARS-CoV-2 and the mortality induced by COVID-19 [59]. Given that T2DM is accompanied by an inherently increased risk of severe disease in case of COVID-19, patients with this condition, among others, were prioritized in the vaccination campaign [59,60].

Current evidence suggests that glycemic control strongly impacts the efficiency of the immune response in patients receiving an mRNA vaccination against SARS-CoV-2 [61]. Indeed, hyperglycemia at the time of vaccination worsens the immunological response, as assessed by the abundance of SARS-CoV-2-neutralizing antibodies and of CD4+ T cells secreting immunomodulatory cytokines after specific stimulation with virus-relevant antigens [61]. On the other hand, the immunological response could be improved by achieving adequate glycemic control during the post-vaccination period [61]. Therefore, stringent glycemic control might improve or even restore a proper immune response to SARS-CoV-2 vaccine [42,60,61]. Thus, it could be relevant and appropriate to improve glycemic control before administering the vaccine to optimize its response, albeit no trial has tested this hypothesis [42,56,59,60,61,62].

## 9. Conclusions

**Figure 1:**
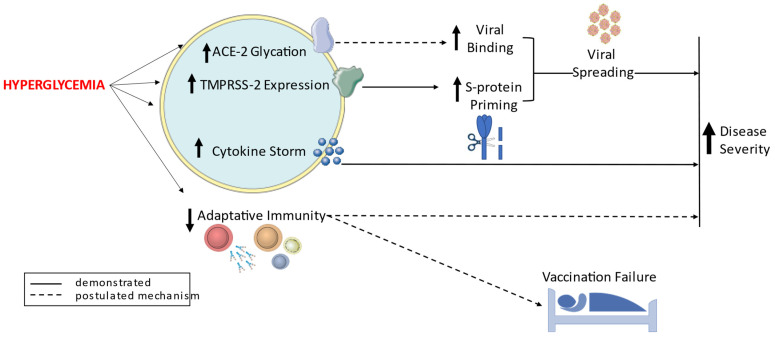
A schematic overview of the proposed mechanisms linking hyperglycemia to COVID-19 related outcomes.

Hyperglycemia, insulin resistance, and metabolic syndrome are emerging as risk factors for poor prognosis and death in patients with COVID-19 [10,13,14,15,16,17,29,30,31,32]. Mechanistically, hyperglycemia can trigger endothelial damage via oxidative stress, over-inflammation (local and systemic), acute thrombosis, and an increase of the SARS-CoV-2 viral spreading and replication (summarized in Figure 1) [2,10,13,14,15,16,17,29,30,31,32]. These effects might mediate the observed noxious impact of hyperglycemia on hard outcomes in COVID-19 patients [10,13,14,15,16,17,29,30,31,32]. Indeed, acute hyperglycemia is a major characteristic of the severe forms of COVID-19, leading to increased mortality, which is also higher in patients with poor long-term glycemic control [10,13,14,15,16,17,29,30,31,32]. Considering also that long-term glycemic control influences the immune responses to vaccination, implementing glycemic control both acute, e.g., using CGM in ICU settings, and chronic, i.e., reaching an HbA1c < 7%, might have an impact on infection rates, disease severity, and eventually death in patients with COVID-19. Clinical trials testing these hypotheses are clearly warranted.

## Data Availability

Not applicable.
